# Evaluation of a novel bicycle helmet concept in oblique impact testing

**DOI:** 10.1016/j.aap.2018.12.017

**Published:** 2019-01-08

**Authors:** Emily Bliven, Alexandra Rouhier, Stanley Tsai, Rémy Willinger, Nicolas Bourdet, Caroline Deck, Steven M. Madey, Michael Bottlang

**Affiliations:** a Biomechanics Laboratory, Legacy Research Institute, Portland, OR, 97232, United States; b Institut de Mécanique des Fluides et des Solides, Université de Strasbourg, France

**Keywords:** Bicycle helmet, Brain injury, Concussion, Impact testing, Impact mitigation, Rotational acceleration

## Abstract

**Background:**

A novel bicycle helmet concept has been developed to mitigate rotational head acceleration, which is a predominant mechanism of traumatic brain injury (TBI). This WAVECEL concept employs a collapsible cellular structure that is recessed within the helmet to provide a rotational suspension. This cellular concept differs from other bicycle helmet technologies for mitigation of rotational head acceleration, such as the commercially available Multi-Directional Impact Protection System (MIPS) technology which employs a slip liner to permit sliding between the helmet and the head during impact. This study quantified the efficacy of both, the WAVECEL cellular concept, and a MIPS helmet, in direct comparison to a traditional bicycle helmet made of rigid expanded polystyrene (EPS).

**Methods:**

Three bicycle helmet types were subjected to oblique impacts in guided vertical drop tests onto an angled anvil: traditional EPS helmets (CONTROL group); helmets with a MIPS slip liner (SLIP group); and helmets with a WAVECEL cellular structure (CELL group). Helmet performance was evaluated using 4.8 m/s impacts onto anvils angled at 30°, 45°, and 60° from the horizontal plane. In addition, helmet performance was tested at a faster speed of 6.2 m/s onto the 45° anvil. Five helmets were tested under each of the four impact conditions for each of the three groups, requiring a total of 60 helmets. Headform kinematics were acquired and used to calculate an injury risk criterion for Abbreviated Injury Score (AIS) 2 brain injury.

**Results:**

Linear acceleration of the headform remained below 90 g and was not associated with the risk of skull fracture in any impact scenario and helmet type. Headform rotational acceleration in the CONTROL group was highest for 6.2 m/s impacts onto the 45° anvil (7.2 ± 0.6 krad/s^2^). In this impact scenario, SLIP helmets and CELL helmets reduced rotational acceleration by 22% (p = 0003) and 73% (p < 0.001), respectively, compared to CONTROL helmets. The CONTROL group had the highest AIS 2 brain injury risk of 59 ± 8% for 6.2 m/s impacts onto the 45° anvil. In this impact scenario, SLIP helmets and CELL helmets reduced the AIS 2 brain injury risk to 34.2% (p = 0.001) and 1.2% (p < 0.001), respectively, compared to CONTROL helmets.

**Discussion:**

Results of this study are limited to a narrow range of impact conditions, but demonstrated the potential that rotational acceleration and the associated brain injury risk can be significantly reduced by the cellular WAVECEL concept or a MIPS slip liner. Results obtained under specific impact angles and impact velocities indicated performance differences between these mechanisms. These differences emphasize the need for continued research and development efforts toward helmet technologies that further improve protection from brain injury over a wide range a realistic impact parameters.

## 1. Introduction

Bicycle helmets are the primary and most effective strategy for head protection during impacts to prevent traumatic brain injury (TBI) (Hoye, 2018). Contemporary bicycle helmets employ a liner of rigid expanded polystyrene foam (EPS) that dampens the impact, reduces the impact force, and in turn reduces linear and angular head accelerations known to cause TBI (McIntosh et al., 2013a). These standard EPS helmets are highly effective in reducing the risk of skull fracture, penetrating injury, and brain injury (Cripton et al., 2014; Fahlstedt et al., 2014; McIntosh et al., 2013b). To further improve protection from rotational TBI, several bicycle helmet designs implement dedicated mechanisms to mitigate rotational head acceleration (Aare and Halldin, 2003; Bland et al., 2018; Fahlstedt et al., 2014; Hansen et al., 2013). These mechanisms generally fall into two categories. The first category employs a spherical slip interface inside the helmet. For example, the Multi-Directional Impact Protection System (MIPS® AB, Täby, Sweden) consists of a thin slip liner that covers the inside of the helmet. This commercially available technology seeks to reduce rotational acceleration of the head by permitting sliding between the helmet and head during impact. The second category employs a collapsible structure that seeks to reduce the shear stiffness of the helmet (Hansen et al., 2013). While not yet commercially available, the present paper evaluates such a collapsible cellular structure that is recessed within the helmet to provide a rotational suspension. This WAVECEL™ cellular structure represents an extension of earlier research by Hansen et al. on an angular impact mitigation system (Hansen et al., 2013).

In common, these mechanisms aim to reduce rotational head acceleration caused by an oblique impact in order to further improve protection from rotational TBI (Sone et al., 2017). The potential benefits of these mechanisms are based on a large body of research, showing that concussions and TBI can readily be caused by rotational head acceleration which subjects brain tissues to shear forces and results in diffuse axonal injury (Gennarelli, 1993; Gutierrez et al., 2001; Holbourn, 2019; Ivarsson et al., 2003; King et al., 1995; Ommaya et al., 2002; Post and Blaine Hoshizaki, 2015; Sahoo et al., 2016).

The majority of real-world “oblique” impacts of helmeted bicyclists occur at impact angles of 30°- 60° degrees (Bourdet et al., 2012, 2014; Otte, 1989). These oblique impacts induce both radial and tangential forces to the head, leading to both linear and rotational head acceleration (McIntosh et al., 2013b; Willinger et al., 2014). The mandatory test of the Consumer Protection Safety Commission (CPSC) for bicycle helmets sold in the U.S. only captures linear acceleration resulting from vertical impacts, whereby the headform is constrained from rotation (CPSC, 1998). Since this CPSC impact-attenuation test does not assess rotational acceleration, it is not suitable to evaluate the efficacy of mechanisms designed to mitigate rotational head acceleration in oblique impacts. Therefore, an advanced helmet impact test method is required to simulate oblique impacts, and to assess the resulting linear and rotational accelerations of a test headform. A wide range of oblique impact test methods have been developed, including impact testing by guided free-fall onto an angled anvil (Bland et al., 2018; Finan et al., 2008; Hansen et al., 2013; Klug et al., 2015; Milne et al., 2013), vertical drops onto a laterally translating impact surface (Aare and Halldin, 2003; McIntosh et al., 2013b; Mills and Gilchrist, 2008) and pendulum impact tests (Bartsch et al., 2012; Rowson et al., 2015). These oblique impact tests frequently employ a Hybrid III 50^th^ percentile male anthropomorphic head and neck combination (Bartsch et al., 2012; Bland et al., 2018; Hansen et al., 2013; McIntosh et al., 2013b; Post and Blaine Hoshizaki, 2015).

The present study employed an advanced helmet impact test method, based on a guided free-fall of a Hybrid III head and neck surrogate, to conduct oblique impact tests and to assess mitigation of linear and rotational head acceleration provided by different helmet technologies. Specifically, this study assessed impact mitigation of prototype helmets with the WAVECEL concept, and commercially available helmets with a MIPS slip liner, in direct comparison to standard EPS helmets for specific impact angles and velocities (Bourdet et al., 2012, 2014). Results of the study were used to test the hypothesis that the mechanisms for impact absorption of WAVECEL and MIPS helmets can provide improved mitigation of rotational acceleration compared to standard EPS bicycle helmets.

## 2. Methods

### 2.1. Helmets

**Fig. 1.**
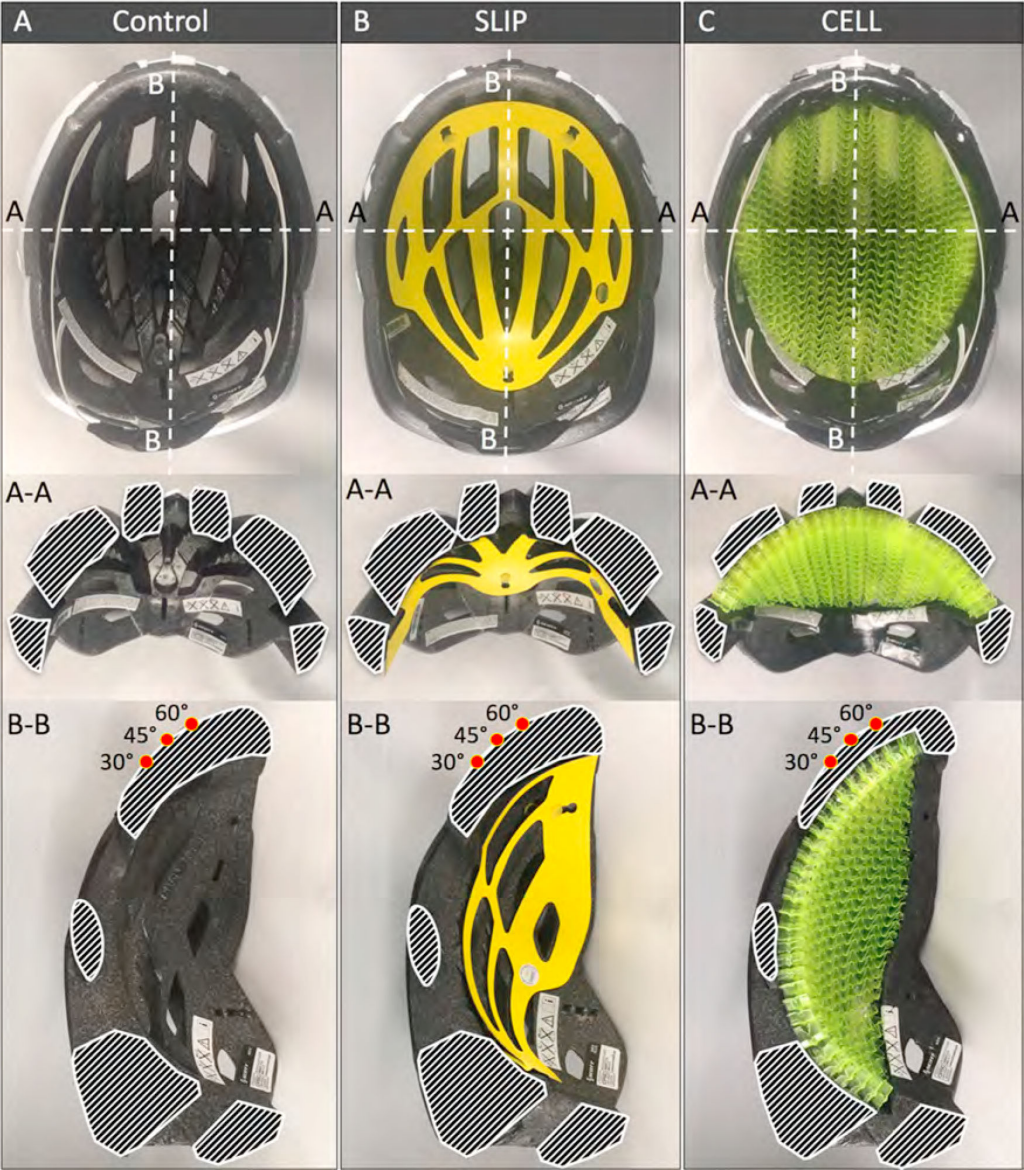
Three helmet types with identical outer shell and liner thickness were tested: A) Standard EPS helmets (CONTROL); B) helmets with a MIPS slip liner for mitigation of rotational acceleration (SLIP); and C) helmets with a cellular structure for mitigation of linear and rotational acceleration (CELL). Sectioned EPS areas along transverse cut (A-A) and sagittal cut (B-B) are outlined in white for illustration. Impact locations corresponding to the 30°, 45°, and 60° anvils are denoted by red dots on sagittal cross-sections. (For interpretation of the references to colour in this figure legend, the reader is referred to the web version of this article).

Three bicycle helmet types were evaluated: standard EPS helmets (CONTROL group), helmets with a MIPS slip liner (SLIP group), and prototype helmets with a WAVECEL cellular structure (CELL group). For the CONTROL group, 20 standard bicycle helmets (Scott ARX, www.scott-sports.com) were tested. These midrange helmets had an in-molded polycarbonate micro-shell and a standard expanded polystyrene (EPS) liner (Fig. 1A). The single-density EPS liner had no embedded reinforcement structures. For the SLIP group, 20 helmets with a MIPS slip liner (Scott ARX Plus, www.scott-sports.com) were tested (Fig. 1B). These helmets were identical to CONTROL helmets, with the exception of the additional slip liner. The Scott ARX Plus was the highest-scoring helmet of Consumer Reports’ 2016 Bike Helmet Ratings (Consumer Reports, 2016). This helmet was selected for the present study to represent a leading mid-priced bicycle helmet. For the CELL group, 20 additional Scott ARX helmets were obtained and modified to implement the cellular WAVECEL structure without affecting the overall thickness of the helmet (Fig. 1C). A 15 mm thick portion of the EPS material was removed by a programmable milling machine from inside of the helmet, leaving approximately 10 mm of the original outer EPS shell. The 15 mm thick cellular structure was placed inside the machined recess to restore the original helmet thickness. At the helmet front, the cellular structure extended approximately 12 mm below the impact line specified in the CPSC impact test for the ISO J headform (CPSC, 1998).

This cellular liner has a specifically designed cell structure to provide distinct mechanisms for absorption of radial and tangential impact forces. For radial impact forces, each cell has a transverse crease to support organized cell buckling. For oblique impact forces, cells can fold in shear direction and the structure can elastically deform in-plane to serve as a rotational suspension between the head and the outer helmet shell. All helmets had the same retention system, outer shell, and overall liner thickness. CONTROL, SLIP, and CELL helmets had an average weight of 208 ± 4 g, 233 ± 6 g, and 282 ± 4 g, respectively.

### 2.2. Test setup

**Fig. 2.**
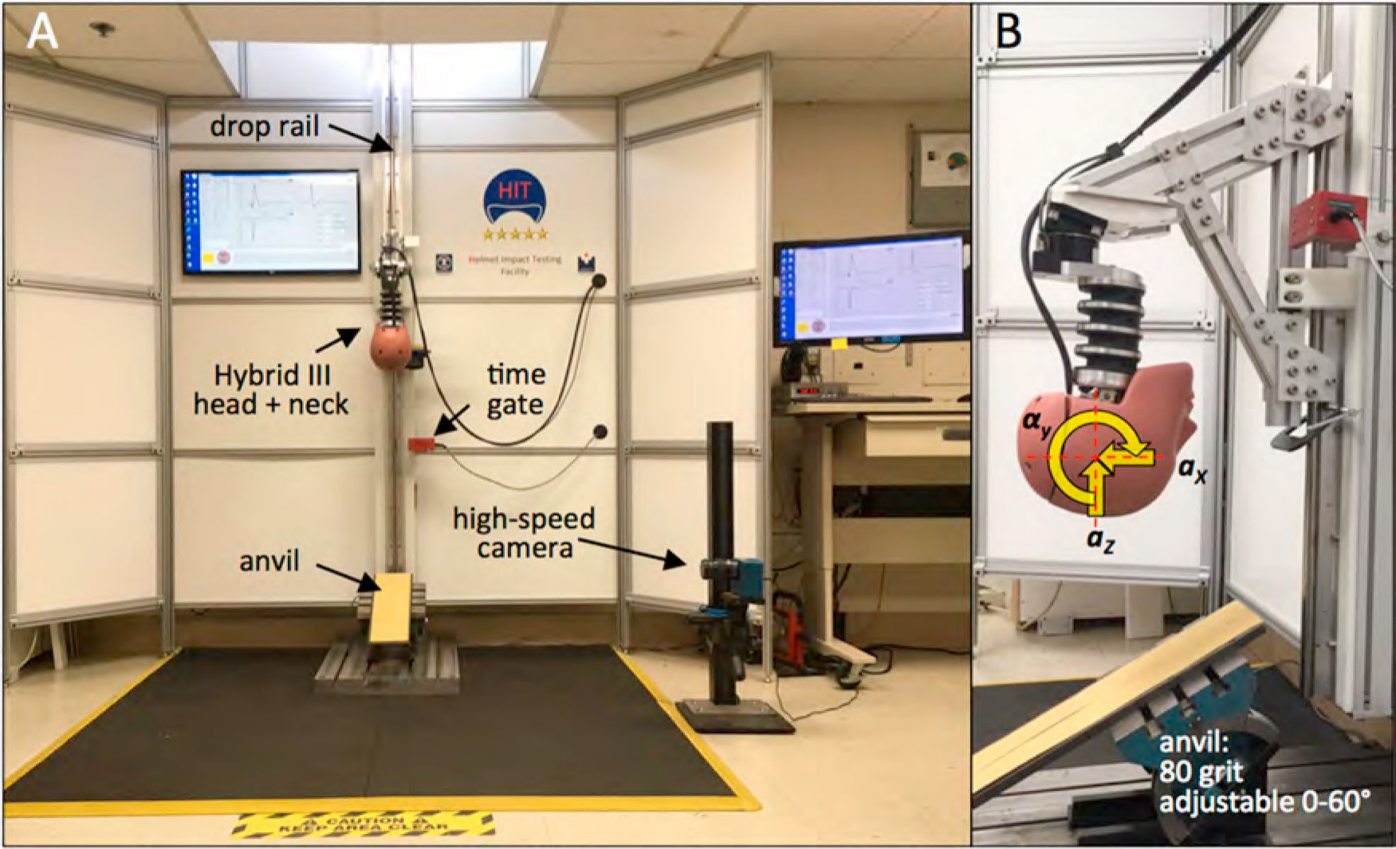
A) Helmet Impact Testing (HIT) facility for vertical drop of a Hybrid III head and neck assembly onto a 0° - 60° adjustable anvil to simulate oblique impacts. B) Drop assembly with linear and rotational headform accelerometers to capture headform kinematics in terms of linear acceleration (*a*) and rotational acceleration (*α*).

Helmet testing was conducted at the Helmet Impact Testing (HIT) facility of the Portland Biomechanics Laboratory (Fig. 2A). In absence of an accepted standard for oblique impact testing of bicycle helmets, the HIT facility was designed to follow recommendations of a recent publication on advanced methods for oblique impact testing (Willinger et al., 2014) and closely corresponds to several published methods of vertical drops onto oblique anvils (Bland et al., 2018; Finan et al., 2008; Hansen et al., 2013). Specific recommendations that were implemented from the publication included: the use of a Hybrid III anthropomorphic headform, which has a more realistic mass and inertia than ISO head forms (Willinger et al., 2014) and which provides a skin cover; (Klug et al., 2015) a Hybrid III neck, which can readily be attached to the headform; (Bartsch et al., 2012; Willinger et al., 2014) assessment of linear and rotational headform acceleration; impact angles in the range of 30°–60° (Bourdet et al., 2012, 2014; Otte, 1989); an impact surface with 80 grit sandpaper according to ECE R-22.05 (ECE, 1999); and inclusion of an impact velocity greater than 6 m/s onto a 45° anvil to better account for real world accident analysis (Bourdet et al., 2012, 2014; Klug et al., 2015; Willinger et al., 2014).

Accordingly, the HIT facility employed a Hybrid III 50^th^ percentile male anthropomorphic head and neck surrogate (78051–336, Humanetic Innovative Solutions, Plymouth, MI) that was connected to a vertical drop tower rail (Fig. 2B). The weight of the drop assembly was 14.0 kg, including the Hybrid III head and neck surrogate and its structural connection to the drop rail, but excluding the helmet. A flat anvil adjustable from 30° to 60° was used to induce oblique impacts in response to vertical drops. Linear head acceleration was captured with a three-axis linear accelerometer (356B21 ICP Triaxial, PCB Piezotronics, Depew, NY) mounted at the center of gravity of the Hybrid III head (Fig. 2B). The resultant linear acceleration *a*_*r*_ was calculated from the three linear acceleration components. Rotational acceleration *α*_*y*_ and rotational velocity *ω*_*y*_ of the headform around the transverse y-axis were measured with a rotational accelerometer (#8838, Kistler Instruments Corp., Amherst, NY). Assessment of headform rotation was limited to rotation around the transverse y-axis, since all impacts were centered onto the sagittal midline of the helmet and the anvil surface was aligned parallel to the headform transverse axis (Hansen et al., 2013). Impact velocity was measured with a time gate (#5012 Velocimeter, Cadex Inc., Quebec, CA).

**Fig. 3.**
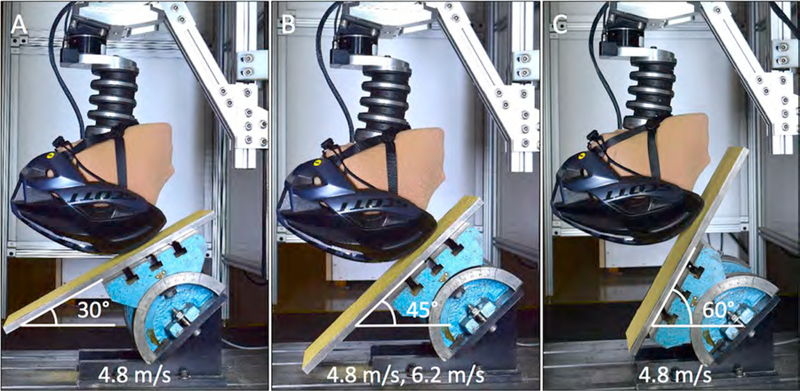
Vertical drop tests of a frontal, mid-sagittal helmet location onto A) a 30° anvil, B) a 45° anvil, and C) a 60° anvil. Anvil angles of 30°, 45°, and 60° correspond to impact angles between the head trajectory and impact surface of 60°, 45°, and 30°, respectively.

Five helmets of each group were tested at 4.8 m/s impact speed on 30°, 45°, and 60° inclined anvils, and additionally at 6.2 m/s on the 45° anvil (Fig. 3). The impact speeds, but not the impact angles, represent those specified in the bicycle helmet safety standard §1203 of the US Consumer Product Safety Commission (CPSC) (CPSC, 1998). As is commonly employed in helmet testing with Hybrid III head surrogates, a double-layer of thin nylon stocking was fitted over the headform to better represent the surface of the human head by reducing the inherently high friction of the Hybrid III silicone scalp (Allison et al., 2014; Jadischke et al., 2016; Pellman et al., 2003; Takhounts et al., 2008). Helmets were properly fitted to the headform with their original fit system. Before each test, new 80 grit sandpaper was applied to the anvil surface (ECE, 1999).

### 2.3. Data acquisition and analysis

Accelerometer data were captured at a sample rate of 20 kHz in a data acquisition system (PCI-6221, National Instruments, Austin, TX). Accelerations were low-pass filtered at Channel Frequency Class (CFC) 1000, as specified by SAE J211 (Instrumentation for impact test, 2007). Rotational velocity ω_y_ was calculated in LabVIEW software using trapezoidal integration of rotational acceleration data.

To estimate the probability of brain injury, the revised Brain Injury Criterion (BrIC) was calculated for each impact, based on peak rotational velocity of the headform (Takhounts et al., 2013). BrIC is an injury criterion based on headform kinematics that was specifically developed for anthropomorphic test devices, including the Hybrid III 50^th^ percentile male head used in the present study. The updated BrIC version provides a critical value (ω_cr_) for rotational velocity around the transverse y-axis of 56.45 rad/s when using a Hybrid III headform (Takhounts et al., 2013). Therefore, BrIC was calculated according to the following equation (Eq. (1)):

(1) $$BrIC=\omega_{y,\;max}/\left(56.45\text{ rad}/s\right)$$

The probability of sustaining an Abbreviated Injury Score (AIS) 2 brain injury was than calculated according to Eq. (2) by implementing the resulting BrIC value into the corresponding brain injury risk correlation, based on maximal principal strain: (Takhounts et al., 2013)

(2) $$P\left(AIS\;2\right)=1-e^{-{\left(\frac{BrIC}{.567}\right)}^{2.84}}$$

A brain injury of severity AIS 2 is defined as a mild-to-moderate concussion with loss of consciousness of less than 1 h (AAAM, 2008).

For statistical analysis, headform kinematics (*a*_*r*_*, α*_*y*_*, ω*_*y*_) and the head injury criterion P(AIS 2) of the SLIP and CELL groups were compared to the CONTROL group using two-sided Student’s *t*-tests and Bonferroni correction for multiple comparisons to test the stated hypotheses. A value of α = 0.05 was used for the evaluation of statistical significance.

## 3. Results

Impact conditions and outcome parameters for each impact scenario and helmet type are summarized in Table 1.

### 3.1. Linear acceleration

**Fig. 4.**
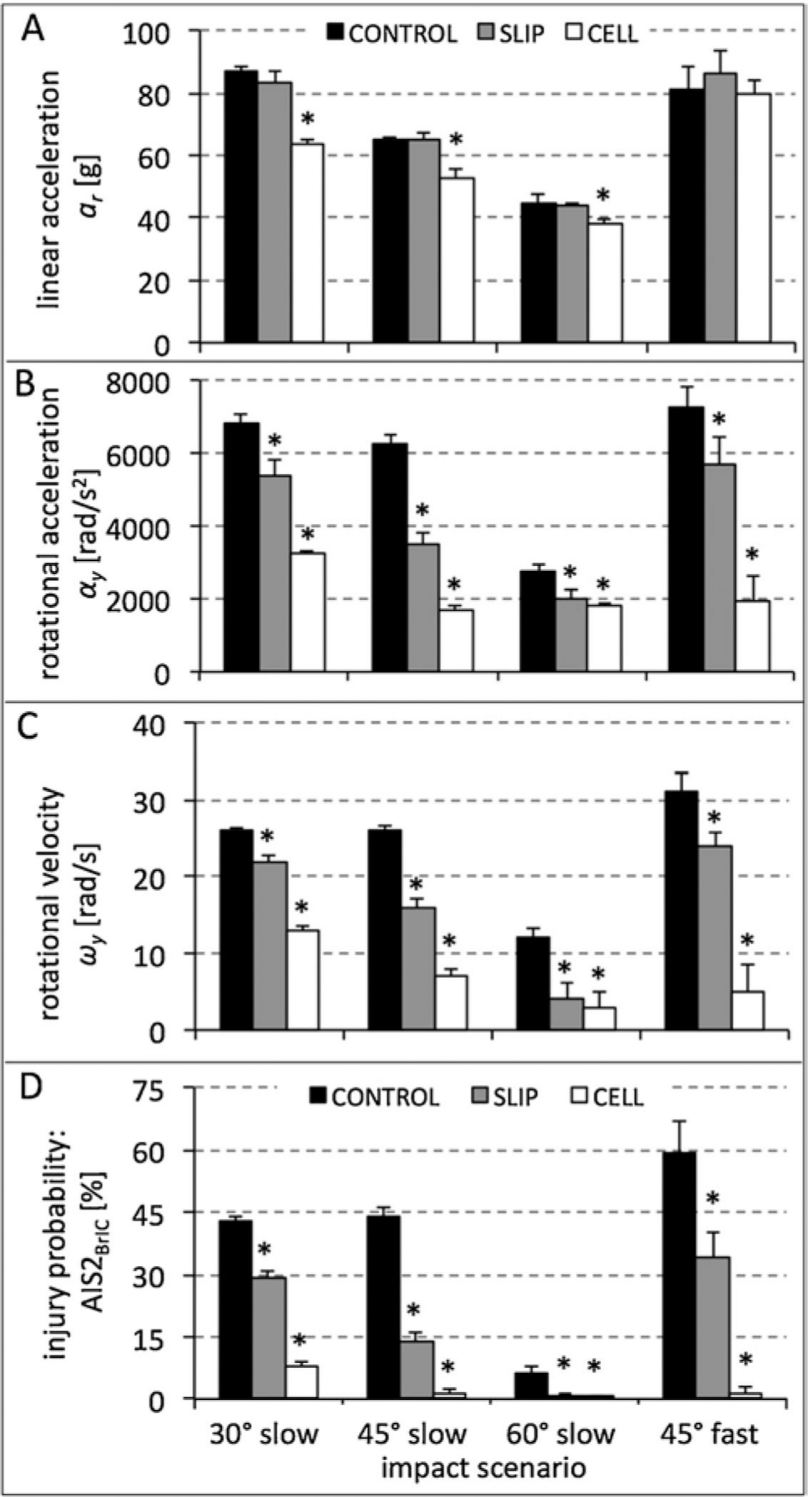
Results for impacts onto the three anvil angles at 4.8 m/s (slow), and for the 45° anvil angle at 6.2 m/s (fast): A) resultant linear headform acceleration,B) headform rotational acceleration, and C) rotational velocity. D) Probability of AIS 2 injury, calculated from peak rotational velocity based on BrIC. (Takhounts et al., 2013) Asterisks denote significant differences (p < 0.05) compared to the CONTROL group.

SLIP helmets did not significantly reduce linear acceleration *a*_*r*_ compared to CONTROL helmets for any impact scenario (Fig. 4A). CELL helmets significantly reduced linear acceleration compared to CONTROL helmets only for slow impacts, with reductions ranging from 16% (60° anvil) to 26% (30° anvil).

### 3.2. Rotational acceleration

SLIP helmets significantly reduced rotational acceleration *a*_*r*_ compared to CONTROL helmets in all impact scenarios (Fig. 4B), with reductions ranging from 21% (30° slow impact) to 44% (45° slow impact). CELL helmets significantly reduced rotational acceleration compared to CONTROL helmets in all impact scenarios, with reductions ranging from 34% (60° slow impact) to 73% (45° fast impact).

### 3.3. Rotational velocity

SLIP helmets significantly reduced rotational velocity *ω*_*y*_ compared to CONTROL helmets in all impact scenarios (Fig. 4C), with reductions ranging from 15% (30° slow impact) to 67% (60° slow impact). CELL helmets significantly reduced *ω*_*y*_ compared to CONTROL helmets in all impact scenarios, with reductions ranging from 50% (30° slow impact) to 84% (45° fast impact).

### 3.4. Brain injury risk prediction

SLIP helmets significantly reduced the probability P(AIS 2) of sustaining AIS 2 brain injury compared to CONTROL helmets in all impact scenarios (Fig. 4D), with reductions ranging from 32% (30° slow impact) to 91% (60° slow impact). CELL helmets significantly reduced *P (AIS 2)* compared to CONTROL helmets in all impact scenarios, with reductions ranging from 81% (30° slow impact) to 98% (45° fast impact).

## 4. Discussion

Results of this study demonstrated the potential of two helmet technologies to reduce the rotational acceleration of a Hybrid III head surrogate compared to a control helmet. The results show potential in reducing the risk of rotational TBI. Furthermore, the results suggest that the efficacy by which the SLIP and CELL technologies provide improved protection depends on the impact angle and impact velocity. Since these findings are limited to a specific combination of impact speeds and impact angles, further investigations are warranted to explore higher impact severities accounting for bicycle falls at higher speeds and for collisions with automobiles.

Results of conventional CONTROL group helmets demonstrated that linear acceleration was effectively suppressed to a maximum of 87 g (30° anvil, 4.8 m/s). This linear acceleration is far below the 300 g linear acceleration threshold mandated by the CPCS safety standard (CPSC, 1998). These results closely correlate with an average linear acceleration of 89 g reported by Bland et al. for oblique impact tests of 10 different helmet models onto a 30° anvil at 5.1 m/s (Bland et al., 2018). In contrast to the fixed vertical orientation of the Hybrid III head and neck assembly in the present study, their head-neck assembly was adjustable about two axes to consistently target specific impact locations on the helmet front and sides. While they employed the same Hybrid III neck than the present study, they used a National Operating Committee of Standard for Athletic Equipment (NOCASE) headform. They reported average rotational accelerations as high as 6.4 krad/s^2^ and 9.5 krad/s^2^ for resultant impact speeds of 5.1 m/s and 6.6 m/s, respectively. Similarly, the present study found rotational accelerations as high as 7.2 krad/s^2^ (45° anvil, 6.2 m/s) for CONTROL helmets, resulting in a 59% probability of AIS 2 brain injury. These results confirm the growing recognition that contemporary bicycle helmets can effectively prevent skull fractures, but may not be as effective in mitigating rotationally-induced brain injury (Sone et al., 2017).

For SLIP helmets, the slip liner had no significant effect on linear headform acceleration, as a slip liner is not designed to mitigate radial impact forces. However, by permitting sliding between the helmet and the head during impact, the slip liner significantly reduced rotational headform acceleration to a maximum of 5.7 krad/s^2^ (45° anvil, 6.2 m/s). This was associated with a significant reduction in the probability of AIS 2 injury compared to CONTROL helmets. In the study by Bland et al., two of the 10 helmet model that were tested contained MIPS slip liners (Bland et al., 2018). These two MIPS helmet models resulted in an average rotational headform acceleration of 6.0 krad/s^2^, while the average rotational acceleration of the 8 helmet models without MIPS liner was 5.3 krad/s^2^. Accordingly, the authors stated that *“the two helmet models containing MIPS did not appear to provide superior protection compared to the non-MIPS helmets”* (Bland et al., 2018). Therefore, while the SLIP group demonstrated significant benefits of MIPS liners relative to standard helmets in the present study, the limited degree by which this slip liner mitigated rotational head acceleration warrants exploration of alternative strategies. Furthermore, the impact performance gains of SLIP group helmets came at the cost of a 12% increase in helmet weight compared to CONTROL group helmets.

CELL group results demonstrated a significant reduction in linear acceleration by up to 26% (30° anvil, 4.8 m/s) compared to the CONTROL group. This finding suggested that controlled buckling of an organized cellular structure may have attenuated radial impacts better than compression of traditional EPS foam (Bland et al., 2018). Cellular honeycomb structures for protective helmets have been previously explored, since they can deliver controlled energy absorption in a lightweight structure that also permits heat transfer and airflow (Caccese et al., 2013; Caserta et al., 2011; Hansen et al., 2013). In the comparison study of 10 bicycle helmet models by Bland et al, the highest-ranked model was the only helmet that incorporated a honeycomb structure (Bland et al., 2018). The finding that CELL helmets did not significantly affect linear acceleration in 6.2 m/s impacts suggests that the compressive stiffness of the cellular liner could potentially be modified to enhance mitigation of radial impact forces over a wider range of impact speeds. More importantly, CELL helmets reduced rotational acceleration to well below 4 krad/s^2^ in all tests. As a result, the probability of AIS 2 injury did not exceed 8%, regardless of the test condition. The observed mitigation of rotational acceleration with CELL helmets can be attributed to two unique features of the cellular structure. First, each cell has a geometric feature that allows the cell to fold on its side in a shear manner as a means to absorb shear loading between the outer helmet shell and the head. Second, the cellular structure can undergo elastic in-plane deformation to provide a rotational suspension that decouples the head from the helmet shell. An earlier attempt of employing a cellular structure as a rotational suspension system in bicycle helmets has been introduced by Hansen et al in the form of an Angular Impact Mitigation (AIM) system, comprised of an elastically suspended aluminum honeycomb liner (Hansen et al., 2013). In vertical drop tests at 4.8 m/s onto a 30° anvil, their cellular structure reduced linear acceleration by 14%, rotational acceleration by 34%, and neck loading by up to 32% compared to a traditional EPS bicycle helmet. In combination, these findings suggest that elastic suspension of a properly designed cellular structure has the potential to reduce rotational acceleration and brain injury risk. These impact performance gains of CELL group helmets came at a cost of a 36% increase in helmet weight compared to CONTROL group helmets. Since helmet weight is critical for consumer adoption, integration of CELL technology into a consumer product should focus on minimizing the associated weight increase. To investigate if CELL helmets can adequately mitigate radial impacts, they were also evaluated in CPSC-compliant impact mitigation tests. Impacts were performed centered on the helmet crown onto a flat anvil at 6.2 m/s (n = 5) and onto a hemispherical anvil at 4.8 m/s (n = 5). Impacts onto the horizontal anvil resulted in 207 ± 2g, and impacts onto the hemispherical anvil resulted in 100 ± 9g. While these results fall short of a formal CPSC impact mitigation test, they further support the feasibility of the CELL concept by demonstrating that CELL prototype helmets mitigated linear acceleration well below the 300 g threshold mandated by CPSC (CPSC, 1998).

Results of this study described the performance of two helmet strategies for mitigation of rotational acceleration in direct comparison to a traditional EPS helmet design, tested at three impact angles and two impact speeds in the same helmet design. Results are therefore limited to these specific study parameters and may not be extrapolated outside the tested parameter range. The test setup and parameters were selected to align as much as possible with established test standards and precedence from similar studies to facilitate reproduction of the test setup in other test facilities. Specifically, impact testing by guided free-fall onto an angled anvil (Bland et al., 2018; Finan et al., 2008; Hansen et al., 2013; Klug et al., 2015; Milne et al., 2013) was chosen over vertical drops onto a laterally translating impact surface (Aare and Halldin, 2003; McIntosh et al., 2013b; Mills and Gilchrist, 2008) or pendulum impact tests (Bartsch et al., 2012; Rowson et al., 2015) for its greater simplicity and high reproducibility (Aare and Halldin, 2003). The Hybrid III 50^th^ percentile male anthropomorphic head was chosen, since it readily allows for sensor integration and Hybrid III neck attachment. It furthermore provides an elastic skin envelope, and its inertial properties are considerably more biofidelic than that of ISO headforms specified in the CPSC safety standard (Willinger et al., 2014). While there is also precedence for impact testing using an unconstrained headform without a neck surrogate (Finan et al., 2008; Klug et al., 2015; Mills and Gilchrist, 2008; Milne et al., 2013), the present study simulated quasi-physiologic head constraints with a Hybrid III neck (Bland et al., 2018). The Hybrid III neck was specifically developed and validated for flexion and extension, but has been shown to be overly stiff in lateral bending (Sances et al., 2002). Moreover, the axial stiffness of the Hybrid III neck has been found to be significantly higher than that of cadaveric neck specimens (Yoganandan et al., 1989). The Hybrid III head and neck combination has been used in a range of helmet impact studies (Bartsch et al., 2012; Bland et al., 2018; Hansen et al., 2013; McIntosh et al., 2013b; Post and Blaine Hoshizaki, 2015) and has been proposed for advanced testing of bicycle helmets (Willinger et al., 2014). The experimental design was limited to impact locations at the helmet front, for which reason results cannot be extrapolated to other impact locations. While the helmet front is the most commonly impacted region, such frontal impacts typically occur at a lateral offset within a 60 ° arc from the mid-sagittal plane (Ching et al., 1997). A mid-sagittal impact location was chosen to simplify the impact kinematics, and to match the impact scenarios in previously published studies (Aare and Halldin, 2003; Finan et al., 2008; Hansen et al., 2013; Ivarsson et al., 2003; Mills and Gilchrist, 2008). While the experimental design was limited to one frontal impact location per impact angle, this impact location shifted toward the helmet rim for the 60° anvil and toward the helmet crown for the 30° anvil since the Hybrid III surrogate retained the same vertical orientation in all impact scenarios. An analysis of 696 retrieved bicycle helmets found that 47% of impacts at the helmet front occurred close to the rim, similar to the 60° anvil test in the present study, and 37% of impacts at the helmet front occurred in the mid-section between the rim and the crown, similar to the 30° and 45° anvil tests (Ching et al., 1997). Impact angles were chosen to represent the 30°- 60° range determined from reconstruction of real-world bicycle accidents (Aare and Halldin, 2003; Bourdet et al., 2012, 2014). The slow (4.8 m/s) and fast (6.2 m/s) impact speeds of the present study align with the impact speeds specified in the CPSC standard for impact testing on curbstone anvils (4.8 m/s) and flat anvils (6.2 m/s) (CPSC, 1998). The 4.8 m/s impacts onto the 30°, 45°, and 60° anvil were comprised of tangential speed components of 2.4 m/s, 3.4 m/s, and 4.2 m/s, and normal speed components of 4.2 m/s, 3.4 m/s, and 2.4 m/s, respectively. The 6.2 m/s impacts onto the 45° anvil were comprised of tangential and normal speed components of 4.4 m/s. These impact speeds are lower than the average impact speeds of 6.4–6.9 m/s reported for helmeted head impacts with a car or the road, which had average tangential and normal speed components of 5.5 m/s and 3.4 m/s, respectively (Bourdet et al., 2012, 2014; McIntosh et al., 2013a). The 14.0 kg weight of the drop assembly in the present study was greater than the 5 ± 1 kg weight requirement for a CPSC drop assembly. However, it was lighter than the drop assembly of Bland et al, in which a 16 kg weight was added to the head and neck assembly to simulate torso mass (Bland et al., 2018).

In addition to limitations due to simplified simulation of real-world impacts under reproducible laboratory conditions, further limitations must be considered when predicting brain injury risk from impact kinematics data. Headform kinematics was analyzed to calculate BrIC from peak rotational velocity. However, prediction of brain injury risk from BrIC depends on the accuracy of injury risk curves that have been reconstructed from a limited number of real-world injury data to estimate brain tolerance limits. Moreover, these injury risk curves are highly non-linear, for which reason a relatively small difference in peak rotational velocity can translate into a large difference in injury probability (Bland et al., 2018). The uncertainty in defining brain tolerance limits combined with the non-linear nature of injury risk curves necessarily limits the accuracy in predicting an absolute probability of brain injury. However, relative differences in brain injury probability between helmet technologies should provide a meaningful comparison, since the helmet technologies were tested in the same helmet model under defined and reproducible impact conditions. Nevertheless, future studies will be required to expand the parameter range of impact conditions, and to include additional helmet technologies.

## 5. Conclusions

Low linear acceleration results suggest that traditional EPS bicycle helmets are highly effective in preventing skull fractures (Cripton et al., 2014; McIntosh et al., 2013b). Conversely, high rotational acceleration results similarly suggest that these helmets have not been optimized to reduce rotational head acceleration in oblique impacts. Since axonal shear strain caused by rotational acceleration is a predominant mechanism of injury in concussions (Meaney and Smith, 2011), strategies for improved helmet designs should therefore target mitigation of rotational acceleration. Results of SLIP and CELL group helmets demonstrated the potential that rotational acceleration of a headform can be significantly reduced by these helmet technologies. Differences in the efficacy between these technologies emphasize the need for continued research and development efforts of helmet technologies that provide improved protection from brain injury over a wide range a realistic impact parameters.

## Figures and Tables

**Table 1:** Summary of results from all impact tests in terms of the average outcomes and standard deviations (STDEV). P-values denote the significance of differences compared to the CONTROL group. “- “ denotes a non-applicable, empty cell.

Outcome	Result	Helmet		30° anvil, slow			45° anvil, slow			60° anvil, slow			45° anvil, fast	
**Cathegory**	**Parameter**	**Type**	**Average**	**STDEV**	**p-value**	**Average**	**STDEV**	**p-value**	**Average**	**STDEV**	**p-value**	**Average**	**STDEV**	**p-value**
	**Impact speed**	CONTROL	4.80	0.02	-	4.81	0.01	-	4.78	0.02	-	6.20	0.02	-
	[m/s]	SLIP	4.82	0.01	0.673	4.79	0.03	0.61	4.80	0.01	0.246	6.15	0.04	0.113
**Impact**		CELL	4.79	0.0	0.559	4.83	0.02	0.551	4.78	0.02	0.811	6.17	0.04	0.259
**Conditions**	**Impact Energy**	CONTROL	163.8	1.4	-	164.2	1.8	-	162.6	1.1	-	272.9	1.5	-
	[J]	SLIP	164.6	1.0	0.673	163.0	1.8	0.612	163.6	0.9	0.246	269.0	3.7	0.113
		CELL	162.8	1.3	0.558	165.3	1.0	0.553	162.0	1.1	0.813	269.9	3.6	0.259
	**lin. acceleration**	CONTROL	87	1.1	-	65	0.7	-	45	2.3	-	81	7.7	-
	*a*_*r*_	SLIP	83	4.3	0.117	65	2.1	0.83	44	1.0	0.997	86	7.8	0.564
	[g]	CELL	64	1.0	< 0.001	53	2.7	< 0.001	38	1.4	0.001	80	4.2	0.808
**Head**	**rot. acceleration**	CONTROL	6821	219	-	6237	255	-	2743	176	-	7243	574	-
**Kinematics**	*α*_*y*_	SLIP	5385	445	< 0.001	3481	359	< 0.001	2023	229	0.001	5683	777	0.014
	[rad/s^2^]	CELL	3262	63	< 0.001	1702	98	< 0.001	1802	98	< 0.001	1962	644	< 0.001
	**rot. velocity**	CONTROL	26	0.3	-	26	0.5	-	12	1.2	-	31	2.5	-
	*ω*_*y*_	SLIP	22	0.7	< 0.001	16	1.1	< 0.001	4	2.2	< 0.001	24	1.8	0.001
	[rad/s]	CELL	13	0.5	< 0.001	7	1.0	< 0.001	3	1.9	< 0.001	5	3.5	< 0.001
**Brain**	**P(AIS2)**	CONTROL	43	1	-	44	2	-	6.4	1.6	-	59.2	8	-
**Injury**	BrIC	SLIP	29	2	< 0.001	14	2	< 0.001	0.6	0.8	< 0.001	34.2	6	0.001
**Risk**	[%]	CELL	8	1	< 0.001	1.2	1	< 0.001	0.2	0.3	< 0.001	1.2	2	< 0.001
